# The Effect of TNF-α on Regulatory T Cell Function in Graft-versus-Host Disease

**DOI:** 10.3389/fimmu.2018.00356

**Published:** 2018-02-28

**Authors:** Antonella Mancusi, Sara Piccinelli, Andrea Velardi, Antonio Pierini

**Affiliations:** ^1^Hematology and Clinical Immunology and Bone Marrow Transplant Program, Department of Medicine, University of Perugia, Perugia, Italy

**Keywords:** TNF-α, regulatory T cells, TNFR2, immune regulation, tolerance, hematopoietic stem cell transplantation, graft-versus-host disease

## Abstract

FoxP3^+^ regulatory T cells (Tregs) are a subset of CD4^+^ T cells that can suppress proliferation and effector functions of T cells, B cells, NK cells, and antigen-presenting cells. Treg deficiency causes dramatic immunologic disease in both animal models and humans. As they are capable to suppress the function and the proliferation of conventional CD4^+^ and CD8^+^ T cells, Treg-based cell therapies are under evaluation for the treatment of various autoimmune diseases and are currently employed to prevent graft-versus-host disease (GvHD) in clinical trials of hematopoietic stem cell transplantation. Even though tumor necrosis factor-α (TNF-α) is well known for its pro-inflammatory role, recent studies show that it promotes Treg activation and suppressive function. In the present review, we discuss the role of TNF-α in Treg function and the possible implications on the actual treatments for immune-mediated diseases, with a particular attention to GvHD.

## Introduction

The recent discoveries of immune suppressive cells such as natural FoxP3^+^ regulatory T cells (Tregs) (1–3), invariant natural killer T cells (4), myeloid derived suppressor cells (5), and others prove the complexity of the mechanisms that underlie the immune response. These findings have prompted studies of the role of immune suppressive cells in different physiologic and pathologic conditions. Tregs are a subset of CD4^+^ T cells that express the alpha chain of the IL-2 receptor (CD25) and a nuclear transcription factor termed forkhead box P3 (FoxP3) (1–3). They can suppress proliferation and function of many other immune cells such as CD4^+^ FoxP3^−^ T cells, CD8^+^ T cells, B cells, NK cells, and antigen-presenting cells. Studies on mouse models and on patients affected by immunodysregulation polyendocrinopathy enteropathy X-linked syndrome, a genetic disease with Treg deficiency due to mutations in *FOXP3* gene, demonstrated that Tregs are required for immune homeostasis and for survival (1–3). These discoveries provided key insights on the cellular mechanisms of immune regulation. Tregs are critical for maintenance of tolerance toward the self in secondary lymphoid organs and peripheral tissues and play an important role in the control of the inflammatory response (1–3, 6). Recently, we and others demonstrated that Tregs can build an immunological niche in the bone marrow for hematopoietic stem cells and B cell precursors allowing for their maintenance and differentiation (7, 8).

As Tregs suppress the function of conventional T cells (Tcons) and other immune cells, Treg-based cell therapies are under evaluation for the treatment of immune-mediated diseases. Recent studies showed that adoptive transfer of Tregs prevents graft-versus-host disease (GvHD), a life-threatening immune complication of allogeneic hematopoietic stem cell transplantation (HSCT). In this setting, donor Tcons mediate alloreactions that eradicate tumor cells [graft-versus-tumor (GvT)], but that are also directed against normal tissues (mainly skin, gut, and liver), causing GvHD (9, 10). Studies in preclinical models and the results of clinical trials prove that infusion of Tcons under the control of Tregs prevents GvHD, while preserving GvT effects (11–14). As Tregs constitute only 1–5% of total peripheral blood CD4^+^ T cells, their paucity and the complexity of their isolation limit further clinical applications. Thus, different strategies have been tested to expand Treg number and/or enhance Treg function *in vitro* and *in vivo* (15).

Tumor necrosis factor-α (TNF-α) is widely known for its pro-inflammatory activity (16–20). In the clinic it is used to enhance immune responses against tumors (21, 22) and several drugs have been developed to limit its function for treating autoimmune diseases (23–28). Its role in the pathogenesis of GvHD has been extensively described: TNF-α is released in patients after conditioning regimens with chemotherapy and/or radiotherapy and during the active phase of acute GvHD, and it is believed to enhance CD8^+^ T cell mediated alloreactivity exacerbating immune destruction of GvHD target tissues (10, 29). Following these studies TNF-α-inhibitory drugs such as the monoclonal antibodies infliximab and adalimumab and the competitive soluble TNF-α receptor etanercept are now in use in the clinic for the treatment of steroid-refractory GvHD (30).

TNF-α is synthetized as a trimeric type II transmembrane protein, which can be cleaved to give rise to soluble extracellular TNF-α. Both membrane and soluble TNF-α are biologically active (16–20). TNF-α can bind two receptors, TNF receptor 1 (TNFR1) and 2 (TNFR2). Membrane TNF-α acts preferentially through TNFR2. TNFR1 is widely expressed on a variety of cells and its engagement triggers pro-inflammatory responses. TNFR2 expression is almost exclusively restricted to immune cells and its binding promotes cell survival and proliferation (17–20). TNFR1 contains a cytoplasmic “death domain,” which recruits the adaptor molecule TNFR1-associated death domain protein (TRADD). TNFR1 interacts with different signaling complexes through TRADD, leading to either cell survival or cell death, depending on cellular context and signaling regulation. TNFR2, that lacks the cytoplasmic death domain sequence, binds directly TNFR-associated factor 2 and activates the nuclear factor “kappa-light-chain-enhancer” of activated B cells (NF-κB) and mitogen-activated protein kinase (MAPK) pathways (19, 20). TNFR1-deficient mice display defects in immunity to infection and in inflammatory response. In contrast, TNFR2-deficient mice show signs of exacerbated inflammation (31). In line with these data, TNFR1-deficient mice are resistant to myelin oligodendrocyte glycoprotein-induced experimental autoimmune encephalomyelitis, that is a model of multiple sclerosis, while TNFR2-deficient mice exhibit more severe disease (32–34). In the same model, TNF-α-deficient mice also show extensive inflammation, demyelination, and high mortality (35). As Tregs preferentially express TNFR2, recent studies explored TNF-α impact on Treg function (36–38). Many of them highlight, not without controversies, the possibility that TNF-α could enhance Treg suppressive activity, suggesting a new regulatory function for TNF-α.

In this review, we will describe how TNF-α impacts on Treg phenotype and function and how Treg immune responses can be modified by TNF-α exposure over time. We will report controversial studies on human Tregs where TNF-α role is not fully elucidated yet. We will also discuss the possible implications of these studies on the actual treatments for immune-mediated diseases mainly focusing on GvHD and we will propose future clinical directions.

## Immune-Regulatory Role of TNF-α in Treg Function

### Role of TNF-α in Mouse Treg Function

The first clear indication of a role of the TNF-α/TNFR2 pathway in Treg function derived from studies in mice (36). *In vitro*, TNF-α in the presence of IL-2 increases the expression of CD25 and FoxP3, enhances the proliferation of Tregs and the suppression they exert on effector T cell proliferation. Mouse Tregs express higher levels of TNFR2 than CD4^+^ CD25^−^ T cells, while both subsets barely express TNFR1 (36). CD4^+^ CD25^+^ TNFR2^+^ Tregs display an activated phenotype (CD45RB^low^, CD62^low^, CD44^high^, high levels of CD69, CD103, GITR, and CTLA-4) and are more suppressive *in vitro* than CD4^+^ CD25^+^ TNFR2^−^ cells (39). *In vitro*, TNF-α in combination with IL-2 selectively upregulates the expression of TNFR2 and other members of the TNF-α receptor superfamily, including OX40, 4-1BB, and FAS on Tregs (40). Studies in TNFR2-deficient mice showed that TNFR2 is required for natural Treg optimal function *in vivo*. In fact, wild-type Tregs controlled colitis induced by the transfer of naïve CD4^+^ T cells into Rag1^−/−^ mice, while TNFR2-deficient Tregs did not (41, 42). Similarly, neutralization of TNF-α exacerbated skin inflammation and was associated with a reduction of Tregs in the draining lymph nodes in a murine model of psoriasis-like disease (43).

Several reports show that Treg activation through the TNF-α/TNFR2 pathway can be exploited to enhance protection from GvHD in mouse models of allogeneic HSCT. In a mouse model of HSCT serum of mice during acute GvHD contained high levels of TNF-α that induced Treg proliferation and suppressive function. Donor TNF-α-primed Tregs prevented GvHD and prolonged mouse survival at an unfavorable Treg:Tcon ratio compared with unprimed Tregs. Importantly, the donor T cell mediated GvT effect against a leukemia cell line was unaffected (44). In another study, donor Treg-mediated protection from GvHD was abrogated by using a TNFR2 blocking mAb, or when either TNFR2-deficient Tregs or TNF-α-deficient T cells were infused (45). Finally, Chopra et al. showed that treatment of irradiated recipient mice with a TNFR2 specific agonist protein successfully expanded radiation-resistant host Tregs *in vivo*, resulting in prolonged survival and reduced GvHD severity after transplantation. The GvT effect and the function of donor T cells against pathogens (e.g., cytomegalovirus) were preserved even after host–Treg expansion induced by the TNFR2 agonist. The beneficial effects of the TNFR2 agonist were abrogated in TNFR2-deficient mice (46).

### Role of TNF-α in Human Treg Function

Like mouse Tregs, human Tregs preferentially express TNFR2 (37, 38). The majority of human CD4^+^ CD25^+^ TNFR2^+^ cells express FOXP3 and high levels of CD45RO, a marker of activated effector and memory T cells. *In vitro*, they suppress Tcon proliferation and function more efficiently than CD4^+^ CD25^+^ TNFR2^−^ cells (38). Okubo et al. showed that TNF-α or a TNFR2 agonist antibody promote *in vitro* expansion of TNFR2^+^ Tregs when added to standard expansion protocols (culturing medium with anti-CD3/CD28 stimulus and IL-2, in the presence or not of rapamycin) (47). TNFR2 stimulated and expanded Tregs had a striking homogeneous phenotype (CD4^+^ CD25^high^ FOXP3^+^ CTLA4^+^ CD127^−^ CD62L^+^ Fas^+^ HLA-DR^+^ CD45RO^+^ CCR5^−^ CCR6^−^ CCR7^−^ CXCR3^−^ ICOS^−^), they were endowed with a greater suppressive function, and produced lower levels of IFN-γ and IL-10. In fact, such highly suppressive Tregs co-expressing TNFR2 could ameliorate the onset of autoimmune diseases. For example, in type 1 diabetic patients the same authors observed an increase in resting CD45RA^+^ Tregs and a decrease in activated CD45RO^+^ Tregs. *In vitro* treatment with TNF-α or TNFR2 agonist antibody corrected the activation defect of Tregs in these patients (48). Thus, TNFR2 activation could trigger survival and proliferation of human Tregs through the NF-κB and MAPK pathways.

Despite the similarities between mouse and human Tregs expressing TNFR2, there are conflicting data on the effects of TNF-α on human Tregs. Some studies suggest that Treg function is impaired in rheumatoid arthritis (RA) patients and treatment with anti-TNF-α antibodies restores it (49–52). Tregs from patients with active RA or with active Systemic Lupus Erythematosus have been showed to express reduced levels of FOXP3 but increased levels of TNFR2 and to have defective function *in vitro* (50, 53). The mechanisms underlying Treg defective function are not well understood and TNFR2 expression levels could not be involved in the pathogenesis of these autoimmune disorders. Moreover, function of Tregs from patients with various autoimmune diseases could have been affected by many factors, including disease status and previous treatments. Furthermore, anti-TNF-α therapy has been shown to be associated with the induction of a population of CD62L^−^-induced Tregs rather than a recovery in natural Treg function (54). In another study, the anti-TNF-α antibody adalimumab was shown to bind to membrane TNF-α expressed by monocytes and to promote Treg expansion by paradoxically enhancing TNFR2-mediated signaling in RA patients (55).

Conflicting data also arose from *in vitro* analyses of TNF-α effects on Tregs from healthy donors. Some studies showed that suppression of Tcon proliferation exerted by Tregs was impaired in the presence of TNF-α (50–52, 56, 57). On the other hand, other authors reported that TNF-α in combination with IL-2 increased CD25 and FOXP3 expression and induced Treg proliferation and function (47, 48, 58). Different experimental conditions could account for these inconsistencies, such as Treg selection methods and purity, length of Treg exposure to TNF-α and its concentration, and TNF-α effects on effector T cells in coculture experiments. Our personal observations support the notion that TNF-α upregulates the expression of Treg specific markers and it does not impair Treg function *in vitro*. However, these contradictory results highlight the need for an extensive investigation of the role of TNF-α in Treg function *in vivo*, in humanized preclinical models.

## Anti-TNF-α Therapies and TNFR2 Pathway Blockade

The intrinsic pro-inflammatory role of TNF-α and its ability to induce production of other inflammatory cytokines (e.g., IL-1, IL-6, GM-CSF, IFN-γ) made TNF-α an ideal therapeutic target for conditions where a reduction of inflammatory response was needed (23–28, 59, 60). Thus, drugs that block or reduce TNF-α activity have been developed to treat autoimmune diseases, such as RA, inflammatory bowel diseases, psoriasis, ankylosing spondylitis, and others. The recombinant anti-TNF-α antibody infliximab, which blocks both soluble and membrane TNF-α, demonstrated clear clinical efficacy in the treatment of Crohn’s disease and RA. Following this initial success several other anti-TNF-α drugs were tested in the clinic and anti-TNF-α treatment is now a fundamental step in the treatment of autoimmunity (25, 61).

Studies on GvHD after HSCT showed that TNF-α levels are increased in patients with acute GvHD and tend to correlate with disease onset and progression (10, 29, 30, 62). TNF-α is rapidly released by tissue macrophages after the conditioning regimen and it induces donor T cell activation and further proliferation possibly triggering GvHD. Thus, anti-TNF-α therapy was rapidly considered in this condition: infliximab and etanercept (a human recombinant TNF-α receptor that competes for and inactivates soluble and membrane TNF-α) have been used to treat steroid-refractory GvHD (30, 63, 64). After initial studies that were suggesting clinical efficacy, the lack of response in a big portion of patients, the high-risk of life-threatening infections that may follow the treatment, and the possibility of GvHD exacerbations or rapid progression after treatment, are limiting their clinical use and leave doubts on their application in this setting (63).

The clinical effects of anti-TNF-α therapy should be reconsidered by virtue of the new insights on TNF-α/TNFR2 pathway in Treg function. As TNF-α inhibition can reduce Treg *in vivo* suppressive function, the potential benefit of the treatment in inflammatory conditions could be limited. Anti-TNF-α treatments are not effective in some of the autoimmune diseases in which TNF-α is involved. Moreover, patients treated with anti-TNF-α drugs can develop other immune-mediated complications (65, 66). In fact, in multiple sclerosis, whose pathogenesis appears to be sustained by TNF-α (67), TNF-α blockade resulted in unexpected disease progression and onset of new lesions with demyelination (68).

**Figure 1:**
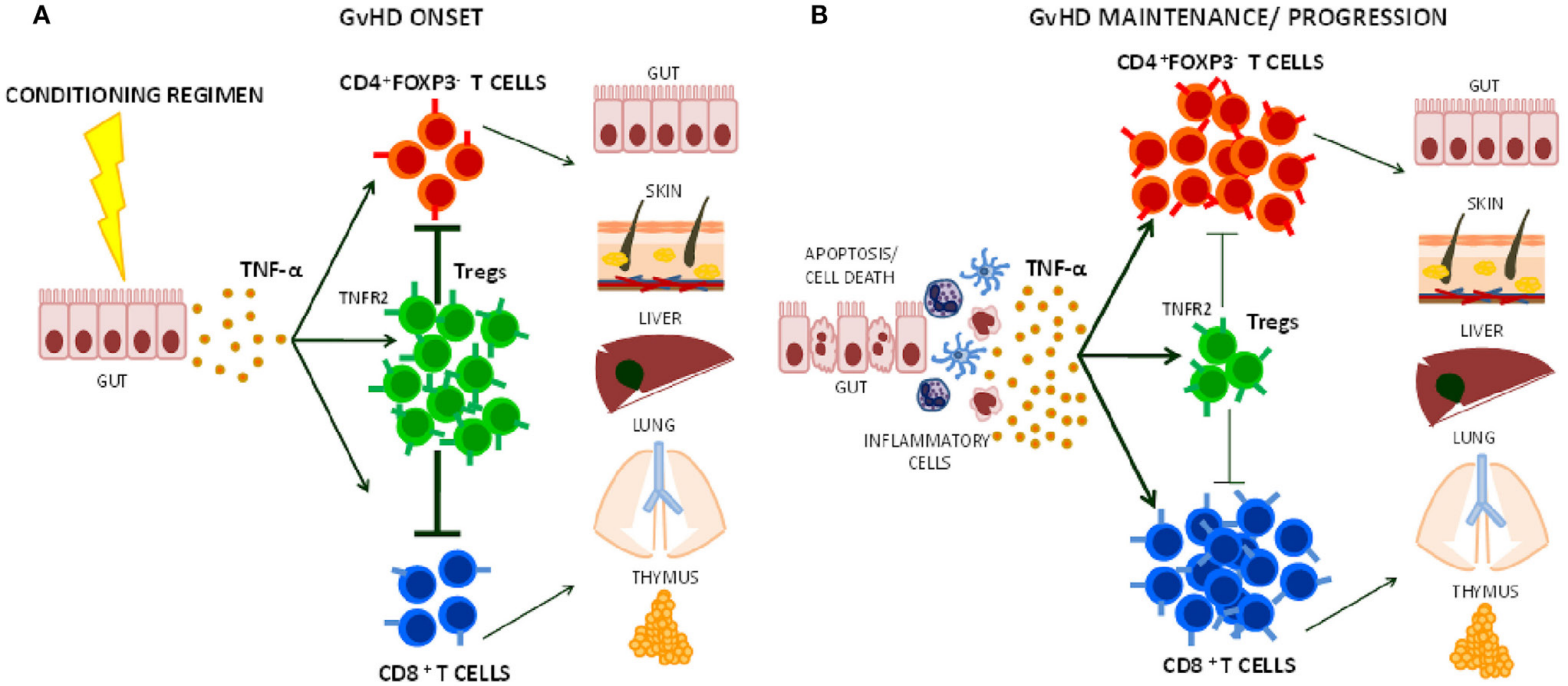
Effects of TNF-α on Treg function during graft-versus-host disease (GvHD) onset, maintenance, and progression. **(A)** The release of TNF-α that follows tissue damage (e.g., gut) due to conditioning regimens with chemotherapy and/or radiotherapy in HSCT induces T cell activation. Its preferential action on Tregs through TNFR2 helps limiting CD4^+^ and CD8^+^ effector T cell function during the early phases of GvHD. **(B)** At later stage, TNF-α may further activate alloreactive T cells contributing to GvHD maintenance and/or progression.

As Tregs are critical for GvHD protection and control over time, limiting Treg function could be a potential pitfall of TNF-α blocking therapy in the HSCT setting. TNF-α that is produced after conditioning regimens with radiotherapy and/or chemotherapy can bind TNFR2 and at the same time activate Tregs and alloreactive T cells (44, 69). The higher TNFR2 expression in Tregs in comparison to the other T cell subsets makes them avid of the cytokine and could favor their activation (Figure 1). Furthermore, Tregs prevent GvHD mainly during the early phase after transplant (15, 70). Thus, the use of anti-TNF-α drugs as GvHD prophylaxis may be particularly counteractive as it could block Treg-mediated suppression of donor alloreactive T cell proliferation in secondary lymphoid tissues (30, 71, 72). Anti-TNF-α drugs are usually used in steroid-refractory GvHD (63). At this stage, TNF-α may have a limited role in sustaining the function of cytotoxic donor alloreactive T cells, which have been already activated and expanded. Moreover, it could be possible that TNF-α blockade limits Treg residual function, thus contributing to disease progression or loss of clinical response in some patients. An optimal window for the use of anti-TNF-α therapy could be the very onset of GvHD when TNF-α recruits and activates donor cytotoxic T cells.

## Clinical Perspectives

Based on the data discussed above, stimulation of the TNF-α/TNFR2 pathway is expected to activate and expand Tregs (36, 38, 39, 44, 47). CD4^+^ FoxP3^+^ Tregs, thanks to their high expression of TNFR2, are preferentially activated when the whole CD4^+^ T cell pool is exposed to TNF-α. In these conditions, they acquire a proliferative and functional advantage in comparison to the effector CD4^+^ FoxP3^−^ T cells suggesting selectivity of the TNF-α/TNFR2 pathway in the CD4^+^ T cell subset (44). Vaccination with Bacillus Calmette Guérin, a strong inducer of TNF-α secretion, promoted in vivo specific expansion of CD4^+^ CD25^high^ FOXP3^+^ Tregs in one subject (47). On the other hand, CD4^+^ CD25^−^ effector T cells upregulate TNFR2 expression after TCR stimulation and become more resistant to Treg-mediated suppression (69). In addition, TNFR2 expression by effector CD4^+^ T cells was required to induce full-fledged experimental colitis in one study (73). Thus, the effects of the activation of the TNF-α/TNFR2 pathway should be carefully evaluated *in vivo*.

Compared with the available anti-TNF-α drugs, blocking antibodies that selectively inhibit TNFR1 or TNFR2 could be used for different clinical purposes (61). Anti-TNFR1 antagonists could be more effective for the treatment of autoimmune diseases, as they do not interfere with Treg function. As Tregs co-expressing TNFR2 are abnormally abundant in human and murine tumors and can support their growth (39, 74), blocking TNF-α/TNFR2 pathway could be a therapeutic option in cancer (75). Indeed, a TNFR2 antagonist antibody has been shown to concomitantly suppress Treg function and promote effector T cell proliferation *in vitro* (76).

**Figure 2:**
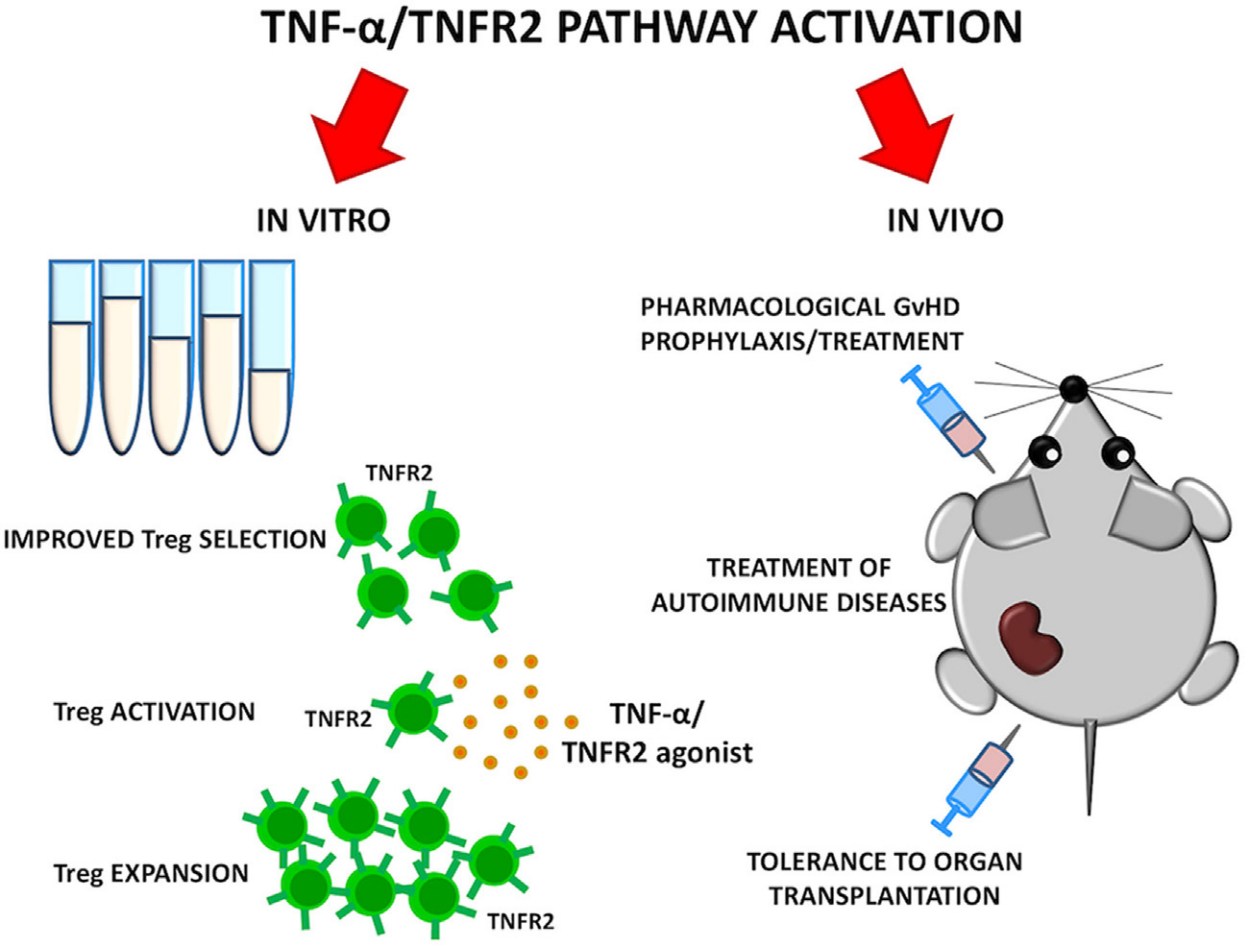
Goals of selective TNFR2 activation on Tregs. TNF-α/TNFR2 pathway could be activated *in vitro* to ameliorate quality of Treg cellular products. Selective TNFR2 agonists may result in preferential Treg activation and expansion *in vivo*. Such strategies could be explored for graft-versus-host disease (GvHD) prevention, treatment of autoimmune diseases, and tolerance induction to organ transplantation.

As TNFR2 is highly expressed by a Treg subset with maximal suppressive function, it could be used as a marker for Treg selection for adoptive therapy purposes. At the same time, treatments that specifically stimulate TNFR2 could selectively boost Treg function. TNFR2 agonists can activate and expand Tregs *ex vivo* and possibly *in vivo* (47). The use of Treg-based cellular therapies is limited by the paucity of Tregs in the periphery and the complexity of *in vitro* manipulation required for their expansion while preserving function and purity. TNFR2 agonists may represent an alternative strategy to expand *in vitro* a Treg population endowed with higher purity and enhanced activity, thus improving results of current Treg-based clinical trials for GvHD prevention in HSCT. Moreover, the ability of TNFR2 agonists to expand highly suppressive Tregs *in vivo* should be carefully evaluated in preclinical models. Such studies could open the possibility of Treg-based immunotherapies for autoimmune diseases where regulation of T cell response is impaired or for tolerance induction to organ-transplantation (Figure 2).

## Concluding Remarks

TNF-α has been widely known for its pro-inflammatory activity, but the effects that follow the stimulation of its two main receptors should be carefully taken in consideration when evaluating the pathogenesis and the treatment of immune-mediated diseases. In this context, new discoveries on the role of TNF-α/TNFR2 pathway may provide relevant tools for a correct use in the clinic of anti-TNF-α treatments and for improving Treg-based therapies. While the blockade of this pathway is under investigation for cancer treatment, TNFR2 stimulation could be used to induce and expand Tregs thus controlling detrimental immune responses. Further studies are needed to evaluate whether Treg activation *via* TNFR2 could enhance yield, purity, and efficacy of Treg-based cell therapies. Such approach could have a potential for quick clinical translation in HSCT trials where Tregs are in use to prevent GvHD and boost immune reconstitution. The rising growth of studies on mouse and human Treg function strongly support a new role for TNF-α and TNFR2 as key players in the complex interplay between immune cells during immune regulation and tolerance.

## Author Contributions

AM, SP, and AP wrote the manuscript. AV provided overall guidance and reviewed the manuscript.

## Conflict of Interest Statement

The authors declare that the research was conducted in the absence of any commercial or financial relationships that could be construed as a potential conflict of interest.
